# Rare Drivers at Low Prevalence with High Cancer Effects in T-Cell and B-Cell Pediatric Acute Lymphoblastic Leukemia

**DOI:** 10.3390/ijms25126589

**Published:** 2024-06-15

**Authors:** Jeffrey D. Mandell, Saathvika Diviti, Mina Xu, Jeffrey P. Townsend

**Affiliations:** 1 Program in Computational Biology and Bioinformatics, Yale University, New Haven, CT 06511, USA; jeffrey.mandell@yale.edu; 2 Biology Department, Colby College, Waterville, ME 04901, USA; sdivit25@colby.edu; 3 Department of Pathology, Yale School of Medicine, New Haven, CT 06510, USA; mina.xu@yale.edu; 4 Program in Genetics, Genomics, and Epigenetics, Yale Cancer Center, New Haven, CT 06520, USA; 5 Department of Biostatistics, Yale School of Public Health, New Haven, CT 06510, USA; 6 Department of Ecology and Evolutionary Biology, Yale University, New Haven, CT 06520, USA

**Keywords:** pediatric acute lymphoblastic leukemia, T-cell, B-cell, genomics, subtypes, therapeutic targets, prevalence, *p* value, cancer effect, personalized medicine

## Abstract

The genomic analyses of pediatric acute lymphoblastic leukemia (ALL) subtypes, particularly T-cell and B-cell lineages, have been pivotal in identifying potential therapeutic targets. Typical genomic analyses have directed attention toward the most commonly mutated genes. However, assessing the contribution of mutations to cancer phenotypes is crucial. Therefore, we estimated the cancer effects (scaled selection coefficients) for somatic substitutions in T-cell and B-cell cohorts, revealing key insights into mutation contributions. Cancer effects for well-known, frequently mutated genes like *NRAS* and *KRAS* in B-ALL were high, which underscores their importance as therapeutic targets. However, less frequently mutated genes *IL7R*, *XBP1*, and *TOX* also demonstrated high cancer effects, suggesting pivotal roles in the development of leukemia when present. In T-ALL, *KRAS* and *NRAS* are less frequently mutated than in B-ALL. However, their cancer effects when present are high in both subtypes. Mutations in *PIK3R1* and *RPL10* were not at high prevalence, yet exhibited some of the highest cancer effects in individual T-cell ALL patients. Even *CDKN2A*, with a low prevalence and relatively modest cancer effect, is potentially highly relevant for the epistatic effects that its mutated form exerts on other mutations. Prioritizing investigation into these moderately frequent but potentially high-impact targets not only presents novel personalized therapeutic opportunities but also enhances the understanding of disease mechanisms and advances precision therapeutics for pediatric ALL.

## 1. Introduction

The comprehensive genomic analyses of the T-cell and B-cell subtypes of pediatric acute lymphoblastic leukemia (ALL) have been performed by tumor sequencing to identify somatic alterations constituting potential therapeutic targets [1,2,3]. In these analyses, genes that were statistically significantly burdened with mutation beyond expectation have typically been ranked by their prevalence in their tumor cohort. This approach directs attention to genes that are most frequently mutated in the patient population, useful knowledge with regard to the potentially treatable population with targeted therapies. However, genetic mutation frequencies (and associated *p* values) do not measure the extent to which mutations contribute to the survival and proliferation of the cancer cell lineages [4]. Low- or moderate-frequency variants are sometimes so central to disease trajectory that they inform molecular subtyping [5]. For the purposes of research prioritization and therapeutic development, the revelation of how much they contribute to cancer phenotypes—their cancer effects—can be highly informative. Scaled selection coefficients provide metrics of cancer effect. Under the population genetic models of cancer evolution, they quantify the extent to which observed substitutions lead to greater cellular proliferation or survival compared to neutral substitutions [6]. Cancer effects are useful indicators of cancer relevance: they are better-performing predictors for the identification of known cancer-related variants compared to mutation prevalence or protein-function impact scores [7].

## 2. Results

**Figure 1:**
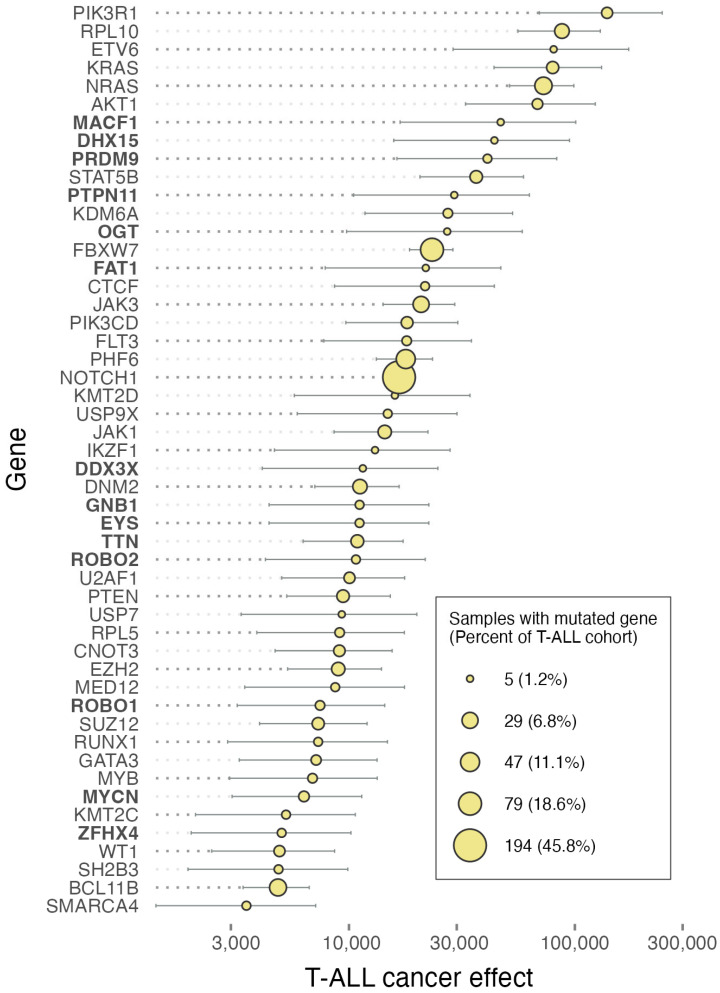
Prevalences of single-nucleotide mutations in genes (circle areas), gene-level cancer effects (population-genetic scaled selection coefficients), and their 95% confidence intervals in pediatric T-cell acute lymphoblastic leukemia based on the Brady et al. T-ALL cohort (*n* = 423) [1]. The highest-effect genes identified as frequently mutated by Brady et al. [1] are listed in roman type. The highest-effect genes that were not identified by Brady et al. [1] are listed in boldface.

We estimated cancer effects for the somatic substitutions reported by Brady et al. [1] in B- and T-cell cohorts using cancereffectsizeR v2.8.0 [7]. Specifically, for each cohort, we measured gene-level cancer effects by estimating a shared scaled selection coefficient across all mutated sites for each gene with greater than four substitutions. In T-cell ALL, the single-nucleotide mutations of *RPL10*, *KRAS*, *NRAS*, *FBXW7*, and *NOTCH1* are at high prevalence and also exhibit substantial to moderate cancer effects (Figure 1). However, the mutations of *PIK3R1* are at considerably lower prevalence yet exhibit the greatest cancer effect. *PIK3R1* encodes a regulatory subunit of PI3K, a key enzyme involved in cell survival and proliferation. Mutations in *PIK3R1* contribute to dysregulated signaling in T-cell ALL, promoting malignant transformation [8]. The mutations of *RPL10* and *NRAS* also manifested at low prevalence in T-cell ALL yet contributed substantial cancer effects. The mutations of ribosomal protein RPL10 enhance JAK-STAT signaling [9], a critical controller of cellular proliferation and survival. Cell lines with an RPL10 R98S substitution exhibit increased sensitivity to clinically relevant JAK-STAT inhibitors like pimozide [9]. *NRAS* mutations activate downstream signaling pathways, promoting uncontrolled proliferation; aberrant NRAS signaling has been shown to foster leukemic cell survival and expansion in T-cell ALL [10].

**Figure 2:**
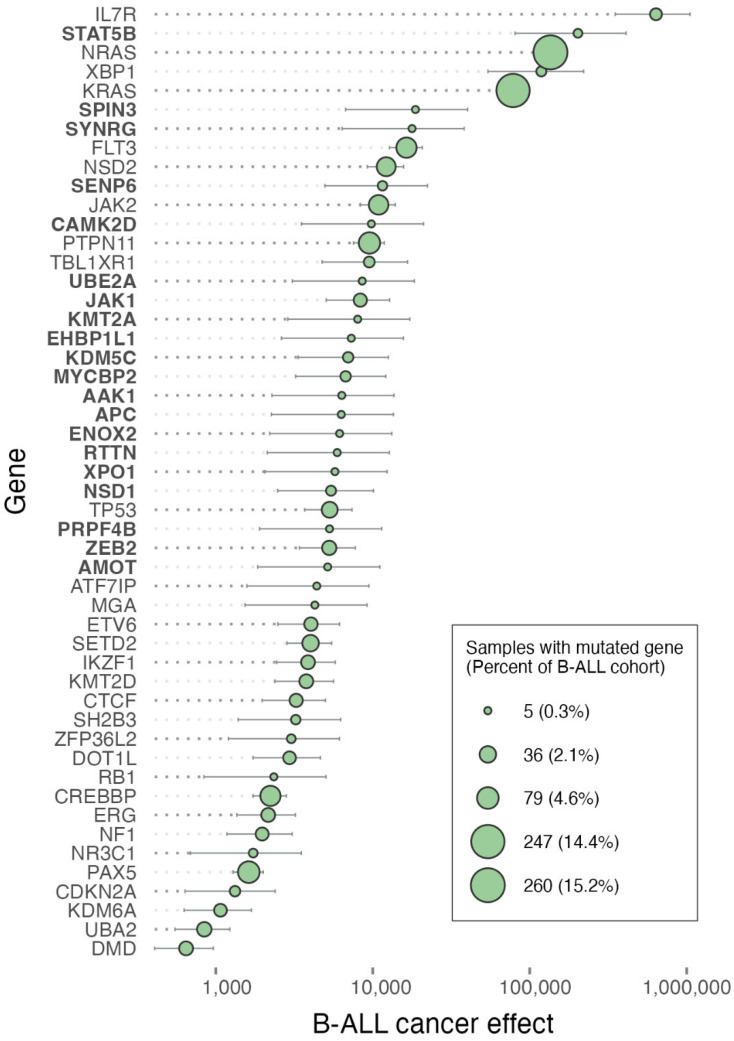
Prevalences of single-nucleotide mutations in genes (circle areas), gene-level cancer effects (population-genetic scaled selection coefficients), and their 95% confidence intervals in pediatric B-cell acute lymphoblastic leukemia based on the Brady et al. B-ALL cohort (*n* = 1712) [1]. The highest-effect genes identified as frequently mutated by Brady et al. [1] are listed in roman type. The highest-effect genes that were not identified by Brady et al. [1] are listed in boldface.

The mutations of *IL7R*, *XBP1*, and *TOX* are at low prevalence in B-cell ALL. Nevertheless, they exhibit some of the highest cancer effects, likely playing crucial roles in the infrequent patients exhibiting these mutations (Figure 2). IL7R plays a crucial role in lymphocyte development and immune system regulation. Mutations in *IL7R* have been associated with altered lymphocyte function, contributing to immune dysregulation, which may have implications for the development and progression of B-cell ALL [11]. XBP1, a key transcription factor involved in the unfolded protein response, is essential for maintaining endoplasmic reticulum homeostasis and proper protein folding [12]. Mutations in *XBP1* may disrupt these processes, potentially leading to cellular stress and contributing to the development of B-cell ALL. Recent molecular biological investigation has revealed a role for TOX in modulating T-cell development and immune responses, indicating emerging connections to leukemia initiation and progression [13]. More commonly associated with T-cell development, the TOX protein is highly expressed in a majority of B-cell lymphomas [14]. Moreover, recent studies have demonstrated the function of TOX in B-cell lineage commitment and differentiation [15], functions that likely underlie its substantial B-cell ALL cancer effect.

Among the well-known, high-prevalence B-cell ALL mutations, those in *NRAS* and *KRAS* are frequently associated with other cancer types such as chronic myelomonocytic leukemia (CMML), particularly the proliferative CMML subtype, which is characterized by a high white blood cell count and poorer prognosis [16]. These mutations play a pivotal role in altering cell growth and differentiation pathways, making them crucial targets for therapeutic interventions [10]. The further development of the inhibitors of RAS remains strongly justified, and their use holds promise for targeted cancer therapy of B-cell ALL, with the potential to disrupt the aberrant signaling pathways and improve treatment outcomes for RAS-mutated patients with these challenging malignancies [17].

## 3. Discussion

Here, we have shown the differential selective impact of mutations within divergent sets of driver genes associated with pediatric T-cell and B-cell acute lymphoblastic leukemia (ALL). By estimating cancer effects for somatic substitutions, we have elucidated their differential genetic landscape, and the extent to which specific mutations contribute to cancer phenotypes, crucial information that provides valuable insights into potential therapeutic targets. Mutations in genes such as *PIK3R1*, *RPL10*, and *NRAS* exhibit substantial cancer effects in T-cell ALL, and are present at low prevalence. However, when present, they appear to be pivotal drivers for malignant transformation and cell survival.

Our analysis reveals that the mutations of *PIK3R1*, a regulatory subunit of phosphoinositide 3-kinase (PI3K), are strong drivers of T-cell ALL. The subunits of PI3K are known drivers in many cancer types [18,19]. Presumably due to the infrequency of its mutation, PI3K has been accorded very little investigation as a driver of T-cell ALL. The small-molecule inhibitors of PI3K have been tested in clinical trials. Historically, they have exhibited limited efficacies as a monotherapeutic agent and a relatively high toxicity [20]. However, preclinical models challenged with recent isoform-selective inhibitors have achieved maximal mutant protein inhibition without eliciting metabolic and glucose homeostasis dysregulation, a major dose-limiting toxicity of PI3K inhibitors [21]. The investigation of the specific effects of the mutations of *PIK3R1* on PI3K activity may reveal vulnerabilities that may be highly effectively targeted in T-cell ALL with these strong cancer driver mutations in *PIK3R1*.

*NRAS* is another proto-oncogene that is frequently mutated in other cancers but relatively infrequently mutated in T-cell ALL. Our analysis provides evidence of its key role in oncogenesis when present in T-cell acute lymphoblastic leukemias. Indeed, NRAS has been shown to cooperate with IL7R to drive T-cell acute lymphoblastic leukemia [22,23,24]. Its presence has also exhibited substantial clinical significance, lowering disease-free survival and rates of complete remission [25]. NRAS, like KRAS and HRAS, has been difficult to inhibit. Recent successes in developing the specific inhibitors of mutant KRAS should encourage renewed attention. Moreover, other strategies have shown potential in melanoma, liver, lung, and gastric cancer, such as targeting NRAS-mutant cancers with a selective STK19 kinase inhibitor [26]. The exploitation of the dependencies of NRAS-mutant tumors on TERT for cellular replicative longevity [27] or on GOLGA7 for NRAS plasma membrane translocation [28] has exhibited promise. The molecular context of these strategies may well be similar in T-cell ALL [29], in which the mutations of *NRAS* exhibit high cancer effect. Therefore, the therapeutic potential of these strategies for tumors with NRAS mutation is substantial.

A more unusual driver of T-cell ALL that nevertheless exhibits high cancer effect is RPL10 R98S, a mutation among several that lead to oncogenic ribosomal lesions [30,31,32,33]. In T-cell ALL, RPL10 R98S mutation promotes the expression of JAK-STAT oncogenes and thereby their oncogenic signaling [9], but also induces elevated oxidative stress [34], a proliferative defect, the promotion of mutagenesis, and the acquisition of rescuing mutations that stimulate proliferation [30]. The net effect of these consequences is a substantial increase in the probability that cancer arises in a cell lineage, resulting in a high cancer effect. This high effect indicates it should receive some attention in the T-ALL community as a therapeutic target, either in synergy with other compounds up front, or specifically in relapsed and refractory settings. All of these strong drivers of T-ALL at relatively low frequencies should be considered in clinically useful precision T-ALL biomarker studies [35], as their high effect indicates the tumor specificity of oncogenic etiology.

The mutations of B-cell ALL that are at high frequency as well as high cancer effect have been identified with poor prognosis and therapeutic outcomes, such as those in *JAK2*, *FLT3*, and genes encoding RAS proteins. These mutations have been associated with Ph-like B-ALL—a poor prognosis group [36]. Mutations in genes such as *IL7R*, *XBP1*, and *TOX* also exhibit substantial cancer effects in B-cell ALL, indicating major roles in the development of respective leukemias. Indeed, their molecular biology substantiates these significant roles. Mutant IL7R has been demonstrated to play a fundamental role in leukemias [22,37,38,39], including B-cell ALL [40]. X-box binding protein 1 (XBP1) is a key regulator of an oncogenic unfolded protein response (UPR) [41,42] with wide-ranging effects that nevertheless plays a crucial, druggable role in pre-B-ALL cell survival, acting downstream of IL7R to support malignant growth via the inhibition of JAK1 and STAT5 [43]; the high expression of *XBP1* confers poor prognosis in ALL patients. TOX has been investigated and shown to play multiple roles in regulating growth, DNA repair, and genomic instability in T-cell ALL [44]. The finding that mutations of these genes play a large role in oncogenesis underscores the importance of deconvolving cohort mutation frequency into underlying mutation and selection when considering therapeutic prioritization in ALL. It is worth noting that because they are infrequent, there is lower statistical power to ascertain whether these high-effect mutations have worse prognoses or therapeutic outcomes.

Our analysis is complemented by highlighting the potential epistatic effects of mutations in genes like *CDKN2A*, emphasizing the need for a comprehensive understanding of the genetic context in therapeutic development. Overall, our results advance the understanding of pediatric ALL pathogenesis and provide a foundation for precision therapeutics targeting high-impact mutations in these subtypes.

As in *PIK3R1*, *RPL10*, *NRAS*, *IL7R*, *XBP1*, and *TOX*, the mutations of other genes identified by their cancer effect can be at low prevalence. Nonetheless, they can have a crucial—even primary—role in disease. Other mutations at low prevalence and even low effect may also be crucially important in a distinct co-occurring genetic context [45,46] or molecularly defined subtype [5,47]; for instance, the single-nucleotide mutations of *CDKN2A* in B-cell and T-cell ALL have a low estimate of effect ignoring context, but the high frequency of *CDKN2A* deletions and their co-occurrence with substitutions (3 out of 5 T-ALL patients with CDKN2A substitutions have the focal deletion of the other copy; among B-ALL patients, 4 out of 12) indicate a large selective epistatic effect. Prioritizing the investigation of these high-impact targets not only opens new avenues for potentially highly effective therapeutic targets but also provides valuable insights into the intricate mechanisms driving the disease, thereby advancing our understanding and ultimately leading to improvements in precision therapeutics.

## 4. Materials and Methods

Data analyzed in this study were obtained from Brady et al. [1], the largest publicly available T-ALL and B-ALL sequence dataset (cf. [48,49,50]). The selection intensity for point mutations was calculated by analysis with cancereffectsizeR version 2.8.0 [7]. Briefly, the expected frequency *μ* that nucleotide mutations occur before they are acted on by selection over the average amount of time elapsed throughout the evolutionary process driving tumorigenesis (from initialization to resection) was determined by calculating the expected frequency that silent mutations occur at the gene level using dNdScv version 0.1.0 [51] hematopoietic cell mutation rate covariates generated from gene expression data from the Cancer Cell Line Encyclopedia [52] and chromatin mark data from Roadmap Epigenomics [53]. Then, nucleotide-level mutation rates were calculated by scaling gene mutation rates in accordance with the trinucleotide-context-specific rates of substitution, such that nucleotide rates within a gene sum to the gene rate. The sample-specific rates of substitution by trinucleotide context were calculated via a mutational signature analysis performed with MutationalPatterns version 3.7.1 [54]. We used COSMIC version 3.2 signature definitions and excluded treatment-associated signatures and signatures presumed absent in ALL [55]. The other details of the calculation, including how rates were calculated for the samples with few mutations, are as previously described [6]. The likelihood of the observed frequency of substitution was maximized based on the underlying mutation rate to determine the selection intensity on each point mutation during the intratumoral fixation process. We composed the likelihood function such that it is consistent with the observation in cancer data that one only observes one selected substitution per site, whereas a flux of mutations at a given rate would generate a Poisson-distributed number of substitutions [56].

## Data Availability

All data analyzed in this study were obtained from the publicly available supplementary materials of Brady et al. [1]. All code used in the analysis is publicly available at https://github.com/Townsend-Lab-Yale/pALL-mutation-effects.
